# A Skull of *Metriorhynchus palpebrosus* in the Kimmeridgian of Normandy (Northwest France) and the Status of the Genus Metriorhynchus: The End of the Mystery?

**DOI:** 10.3390/life16091396

**Published:** 2026-08-24

**Authors:** Stéphane Hua, Jérôme Tabouelle

**Affiliations:** 1 Odyssée Villers Paléospace, Avenue Jean Moulin, 14640 Villers-Sur-Mer, France; 2 Fabrique des Savoirs, 7 Cours Gambetta, 76500 Elbeuf, France; jerome.tabouelle@metropole-rouen-normandie.fr

**Keywords:** *Metriorhynchus palpebrosus*, Kimmeridgian, *Metriorhynchus* definition, taxonomy

## Abstract

This article provides a description of a skull assigned to *Metriorhynchus palpebrosus* discovered in the last century in the Upper Jurassic of Normandy. A reappraisal is made of the Upper Jurassic genus *Metriorhynchus* in Europe and especially of *M. brevirostris* (a type species of the genus), *M. hastifer* and *M. palpebrosus*, which are found in Kimmeridgian levels of France and England. A link is made to synonymize *M. brevirostris* with *M. hastifer*, thanks to a morphological autapomorphic character forgotten since 1886. It allows for the assignment of *M. brevirostris*, a type species of the genus, only known by an isolated snout. This interpretation permits a better understanding of this genus and, by way of the European Upper Jurassic species, a new diagnosis is proposed.

## 1. Introduction

This nearly complete crocodilian skull was illustrated for the first time in 2008 in [1] despite being discovered in the 19th century in the Kimmeridgian of Le Havre (Normandy, northwest France), specifically the Kimmeridgian of Cap de la Hève. In 1859, Pouchet [2] mentioned a nearly complete crocodilian skull in the Beauvoisine Museum of Rouen, and it appears to be one of the rare specimens to have survived the heavy bombing of this area during the liberation of 1944. It was listed as part of the inventory of this museum in 1994 [3]. A number of casts were made in the former “Laboratoire de Paléontologie des Vertébrés de Paris VI” and offered to Le Havre Museum, among others.

**Figure 1:**
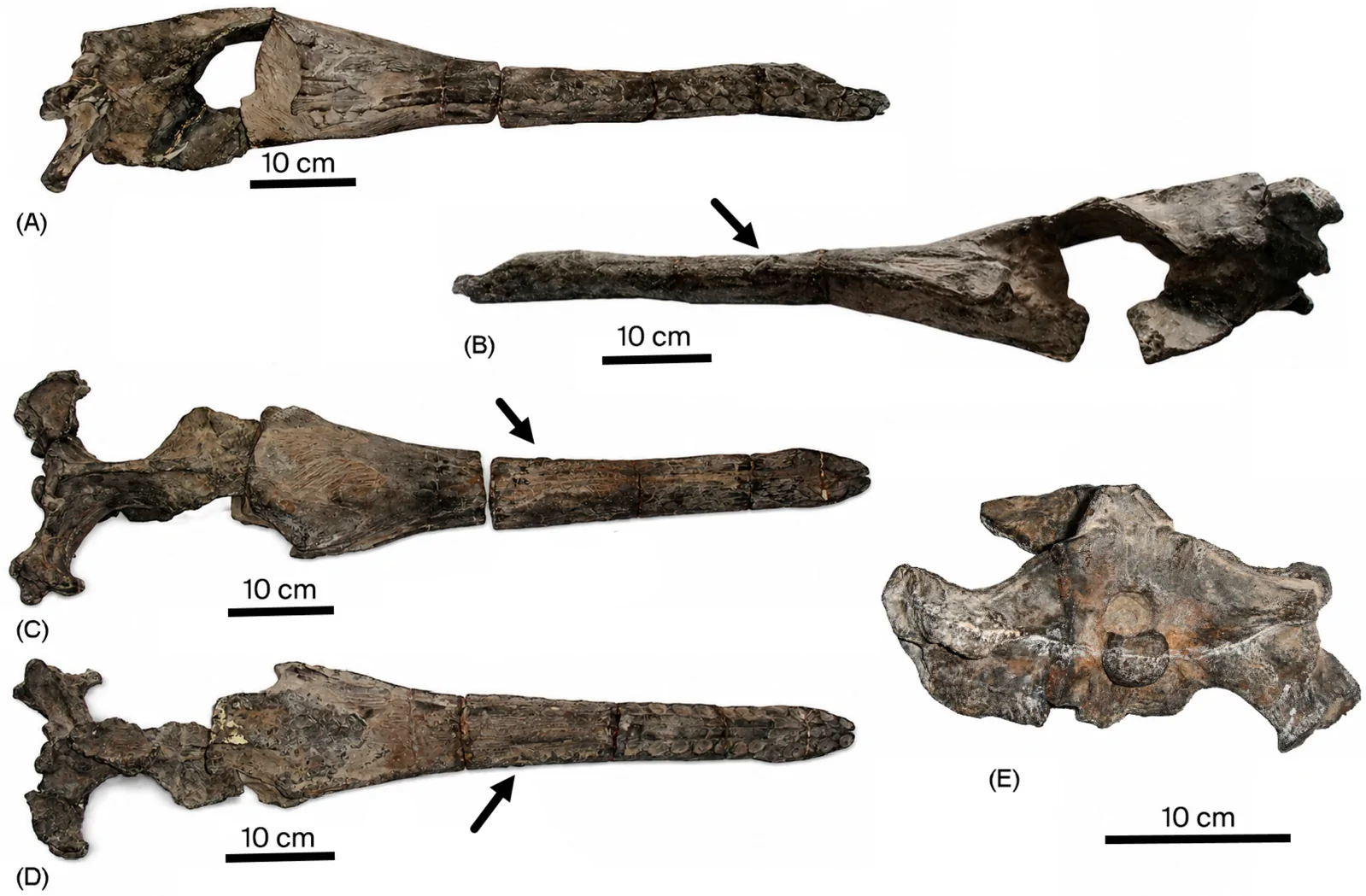
*M. palpebrosus* PAL2008.0.317 (**A**–**E**); scale bar = 10 cm. (**A**) Right lateral view; (**B**) left lateral view, with arrow indicating the abnormal “lateral teeth”; (**C**) dorsal view, idem as previously for the arrow; (**D**) ventral view, idem as previously for the arrow; (**E**) occipital view.

**Figure 2:**
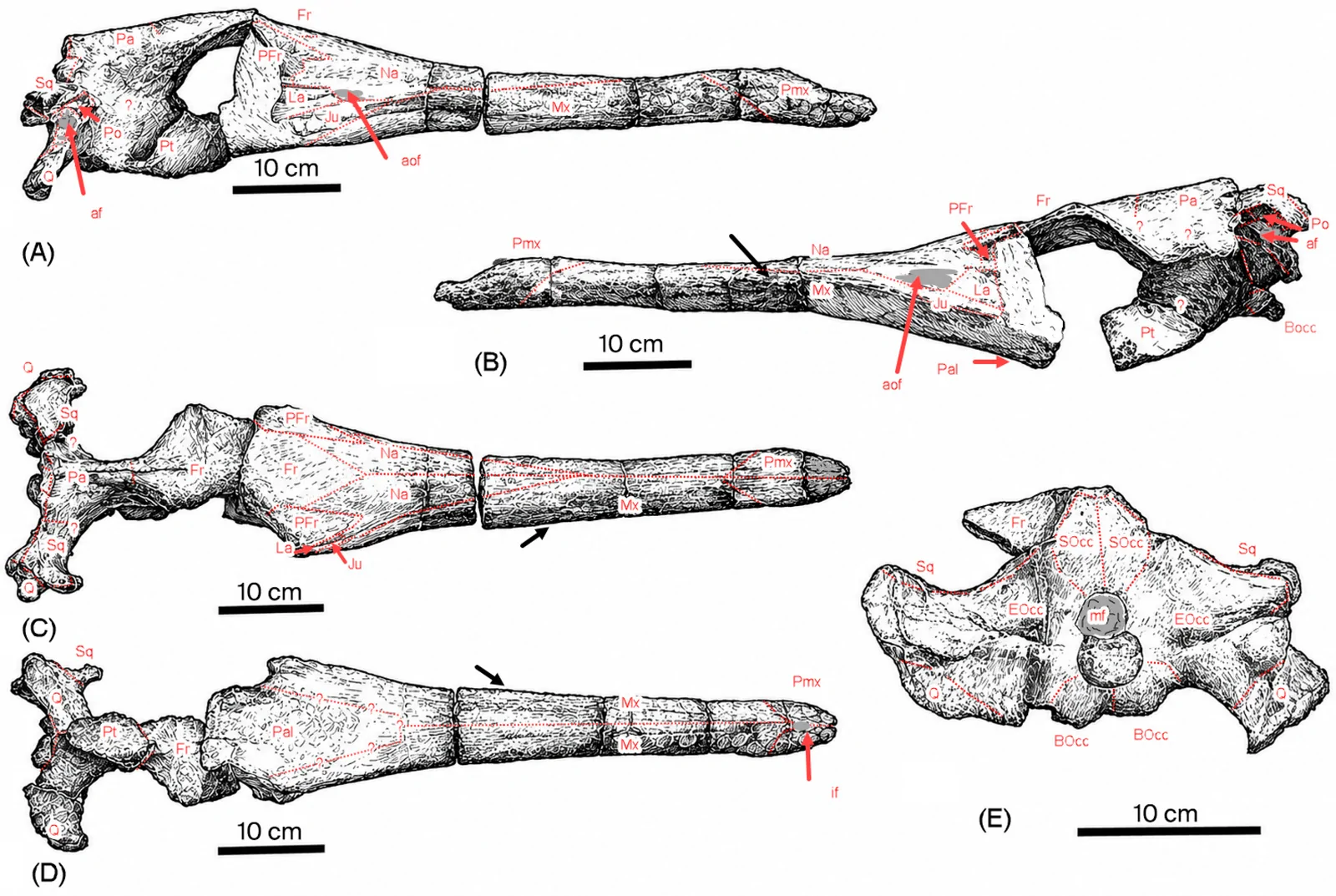
*M. palpebrosus* PAL2008.0.317 (**A**–**E**); scale bar = 10 cm. (**A**) Right lateral view; (**B**) left lateral view, with arrow indicating the abnormal “lateral teeth”; (**C**) dorsal view, idem as previously for the arrow; (**D**) ventral view, idem as previously for the arrow; (**E**) occipital view, indicating sutures and bones in red dotted line, question marks indicating possible suture location. Legend: Bocc: basioccipital; EOcc: exoccipital; Ju: jugal; La: lachrymal; Mx: maxillary; Na: nasal; Fr: frontal; Pal: palatin; Pa: parietal; PFr: prefrontal; Po: postorbitary; Pmx: premaxillary; Pt: pterygoid; Q: quadrate; Socc: supraoccipital; Sq: squamosal; af: auditive fenestra; aof: anteorbital fenestra; if: incisive foramen; mf: magnum foramen.

The skull, which is in three parts, is housed in the Beauvoisine Museum of Rouen (France, Seine-Maritime) under the registration numbers PAL2008.0.317.1, 2 and 3 (Figure 1 and Figure 2). Initially assigned to *Metriorhynchus* cf. *palpebrosus*, (Metriorhynchidae, Thalattosuchia), a family of marine crocodilians distributed worldwide, the genus itself is especially known by various specimens across Europe on the same level from the Callovian to the Kimmeridgian. The number of specimens over two centuries has implied that many authors describe them across many species, with most of them been synonymized since their discovery [4]. This species, *M. Palpebrosus*, initially identified in England, allows us to make a statement regarding this species.

## 2. Systematic Paleontology and Description

The presence of a secondary palate composed of maxillae and palatines allows this specimen to be classified as Crocodylomorpha [5,6].

The prefrontals—which overhang the orbits and are larger than the lachrymals—the laterally placed orbits, the enlarged frontal bone and the lack of an external mandibular fenestra together allow for an attribution to Metriorhynchidae [7,8,9,10,11].

The attribution to the genus *Metriorhynchus* will be explained in a discussion in Section 3, and a specific attribution is provided in Section 3.2.

Order: Mesoeucrocodylia Whestone and Whybrow, 1983 [12];

Sub-order: Thalattosuchia Fraas, 1901 [13];

Family: Metriorhynchidae Fitzinger, 1843 [14];

Genus: *Metriorhynchus* Meyer, 1832 [15];

Species: *Metriorhynchus palpebrosus* (Philips, 1871) [16].

### 2.1. General State of Preservation

**Figure 3:**
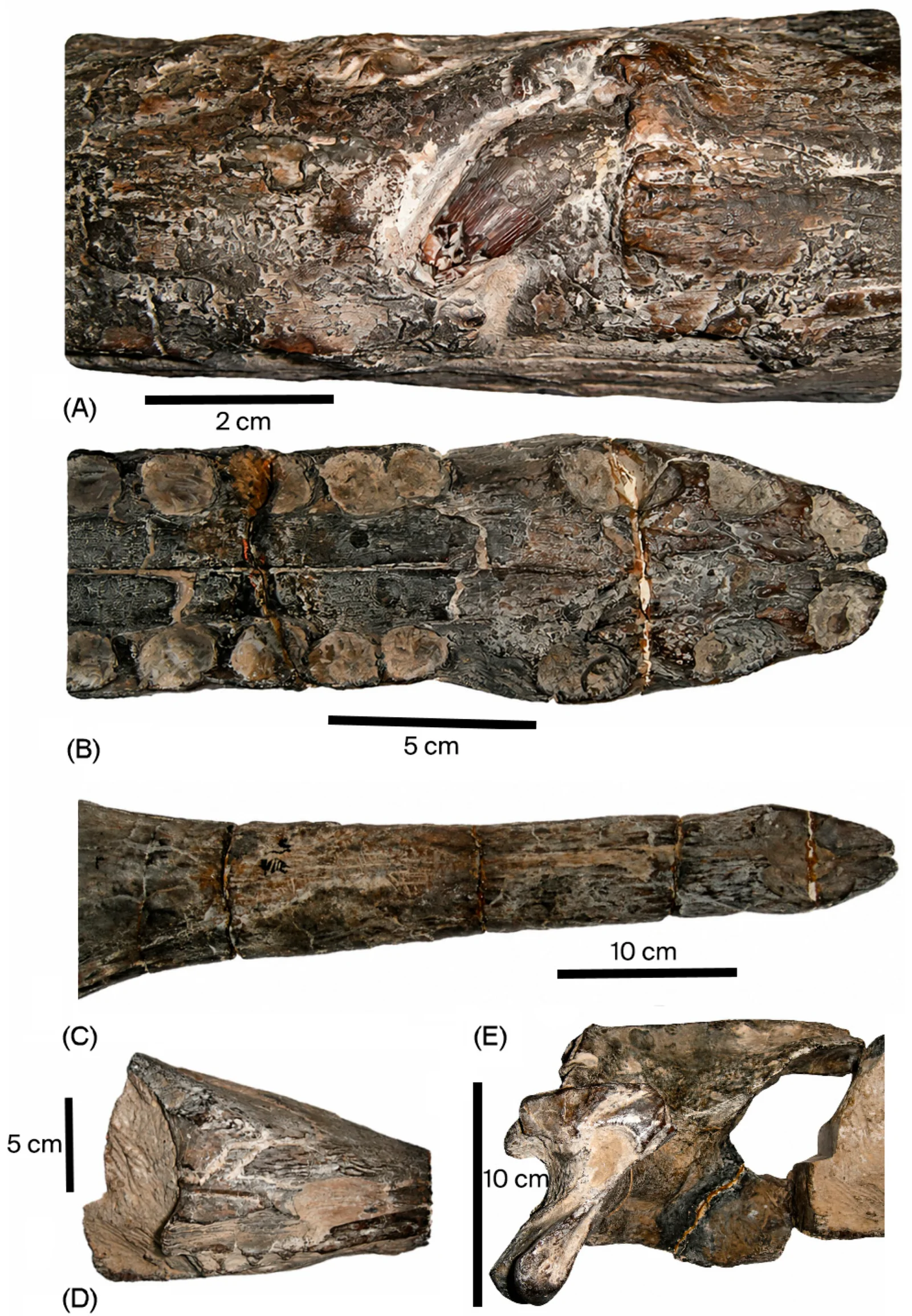
*M. palpebrosus* PAL2008.0.317 (**A**–**E**). (**A**) Detail of the left abnormal lateral teeth (scale bar = 2 cm); (**B**) Ventral view of the rostrum showing the asymmetry of the alveolar rows (scale bar = 5 cm); (**C**) Dorsal view of the snout (scale bar = 10 cm); (**D**) Detail of the right anteorbital area (scale bar = 5 cm); (**E**) Right lateral view of the postorbital area (scale bar = 10 cm).

**Figure 4:**
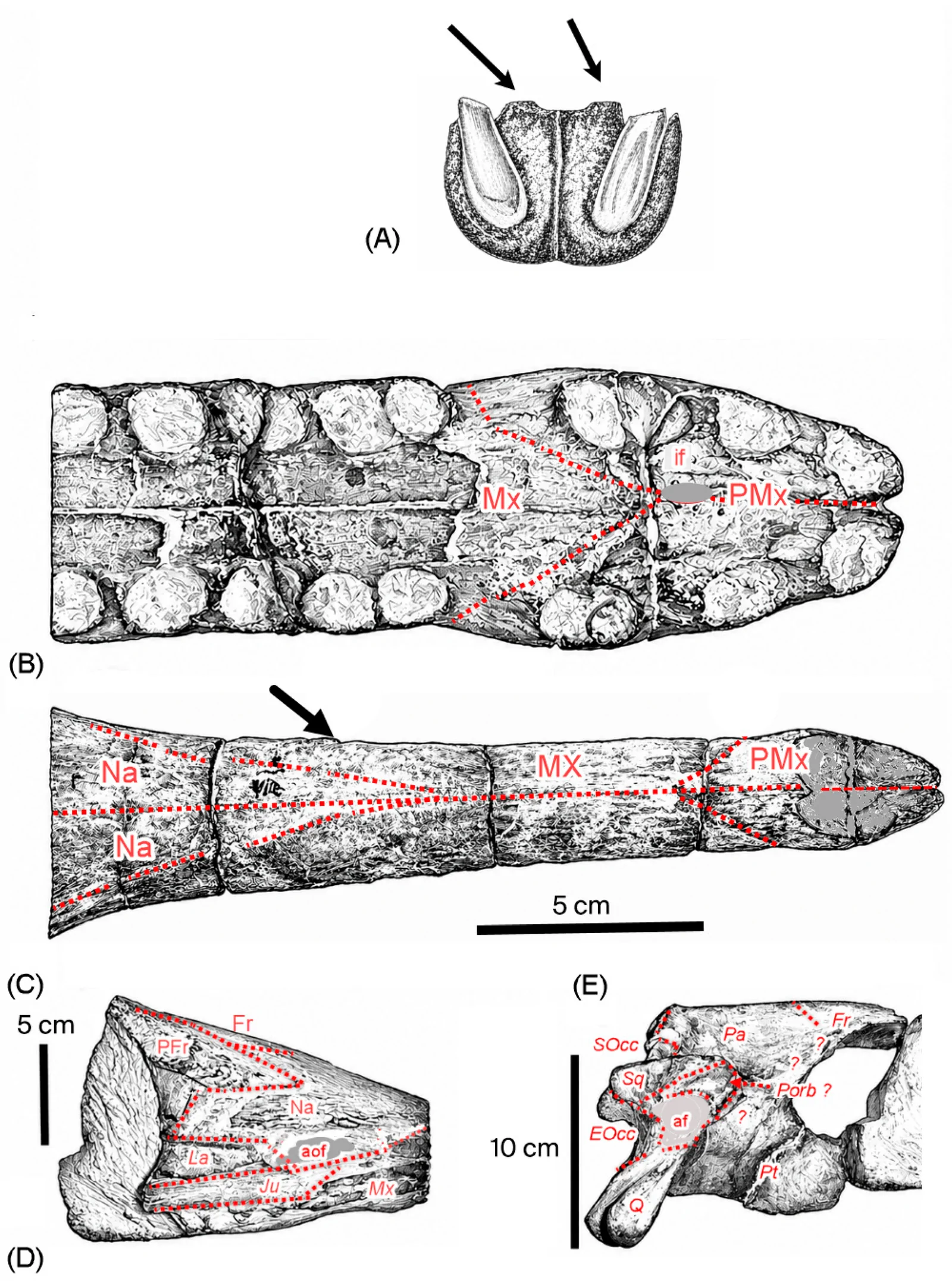
*M. palpebrosus* PAL2008.0.317 (**A**–**E**). (**A**) Modified after Lennier, (1887) pl. XV [19], Figure 3 of a cross-section of the snout of Metriorhynchus brevirostris showing longitudinal convex gutters behind the alveolar rows (indicated by arrows). (**B**) Ventral view of the rostrum showing the asymmetry of the alveolar rows (scale bar = 5 cm); (**C**) Dorsal view of the snout (scale bar = 10 cm); (**D**) Detail of the right anteorbital area (scale bar = 5 cm); (**E**) Right lateral view of the postorbital area (scale bar = 10 cm), with sutures in dotted red lines. Legend: EOcc: exoccipital; Ju: jugal; La: lachrymal; Mx: maxillary; Na: nasal; Fr: frontal; Pa: parietal; Pal: palatin; PFr: prefrontal; Porb: postorbitary; PMx: premaxillary; Pt: pterygoid; Q: quadrate; SOcc: supraoccipital; Sq: squamosal; af: auditive fenestra; aof: anteorbital fenestra; if: incisive foramen; mf: magnum foramen.

This skull is nearly 82 cm long and is broken transversally at two different points; the three parts have been registered as Pal 2008.0.317. 1 to 3 (the snout, the frontal area and postorbital area; Figure 1, Figure 2, Figure 3 and Figure 4).

The skull belongs to an older adult specimen (cranial sutures are totally closed), and it clearly lay exposed on the seafloor for a long time, as evidenced by the many small oyster shells incrusted on both sides of the skull. The body length can be estimated at over 4 m (according to [17] and personal observation of related Callovian skeleton specimens).

The skull here, described dorsoventrally, is tall, strongly contrasting with the compressed aspect exhibited by *M. superciliosus* and other Callovian species of the genus.

This specimen can be considered as longirostrine according to Busbey’s classification [18], with the anteorbital length representing 69% of the total skull length.

The 12th maxillary alveolus is abnormal (Figure 3A): still retaining its tooth, it opens laterally and forward (instead of opening into the buccal cavity). The tooth row is closed after this level due to this pathology. The specimen is an old adult with barely visible, or frequently invisible, sutures.

Total length: 82 cm from the nose to the occipital condyle and 84.5 cm to the external condyle of the quadrate.

Anteorbital length: 56 cm.

Width of the snout: 6.6 cm at the level of the external nare; 7 cm at mid-length; 15 cm (7.5 cm × 2) at the anteorbital rim.

External width: 23.7 cm at the squamosal, 17.3 cm at the interparoccipital process and 24 cm (12 cm × 2) at the inter quadrates (external condyle).

Height: 3 cm at the anterior border of the external nare; 5.2 cm at the posterior.

Height at the level of the pathology: 5 cm.

Height at the level of the frontal area: 13 cm.

Maximum height with the pterygoid wings (fronto-parietal suture): 15.2 cm.

Height of the occipital face (basioccipital apophysis to parietal): 13 cm.

### 2.2. Cranial Apertures (Figure 2, Figure 3 and Figure 4)

External nare: Well preserved; from above, it is heart-like in shape due to the insertion of the internasal septum (preserved for a length of 1 cm) and is elongated. Laterally, the external nare starts just after the level of the first maxillary teeth (and not at the level of the third premaxillary teeth as in the case of the *M. brevirostris* holotype [4] and see the Figure 8 inside). Moreover, the external nare is more elongated (6.8 cm) in the antero-dorsal direction than laterally (4.4 cm). The shape of this feature contrasts with that of the holotype illustrated in [4], which is far more rounded, although it is reconstructed. The anterior border appears open due to the non-closure of the anterior border of the premaxilla.

Incisive foramen: Anteriorly start at the mid-anterior level of the second premaxillary alveolus and not the third alveolus as in the case of *M. brevirostris* type species ([4], Figure 1).

Anteorbital foramen: Can be described on the right side; it is well pronounced but typical of metriorhynchids ([8], the Text-Fig-57), with the non-participation of the prefrontal area.

Orbits: The skull is broken at this level, but like in all metriorhynchids, the orbits are laterally orientated. Despite the poor state of preservation, we can see that again, like in all other metriorhynchids, they are surrounded by the frontal, prefrontal, nasal and lachrymal bones (turning clockwise from the dorsal). The maxillary bone does not participate, and the jugal is too badly damaged to be considered.

Upper temporal fenestra: These can be estimated due to the preservation of the occipital face; they have a trapezoidal shape with a medial length of 16 cm, posterior length of 18 cm and a postero-external to antero-internal length of 19.2 cm.

Foramen magnum: Has a semi-circular shape, with a width of 3 cm and a height of 2.6 cm.

### 2.3. Dentition

There are three premaxillary teeth plus twelve in the left maxilla (due to the pathology), while there are at least 23 alveoli (3 Pmx + 12 Mx alv.) on the right side.

The interalveolar septum appears thinner than in the holotype of *M. brevirostris,* which displays an interspace of half an alveolus, whereas here, we have no more than one-third.

**Figure 5:**
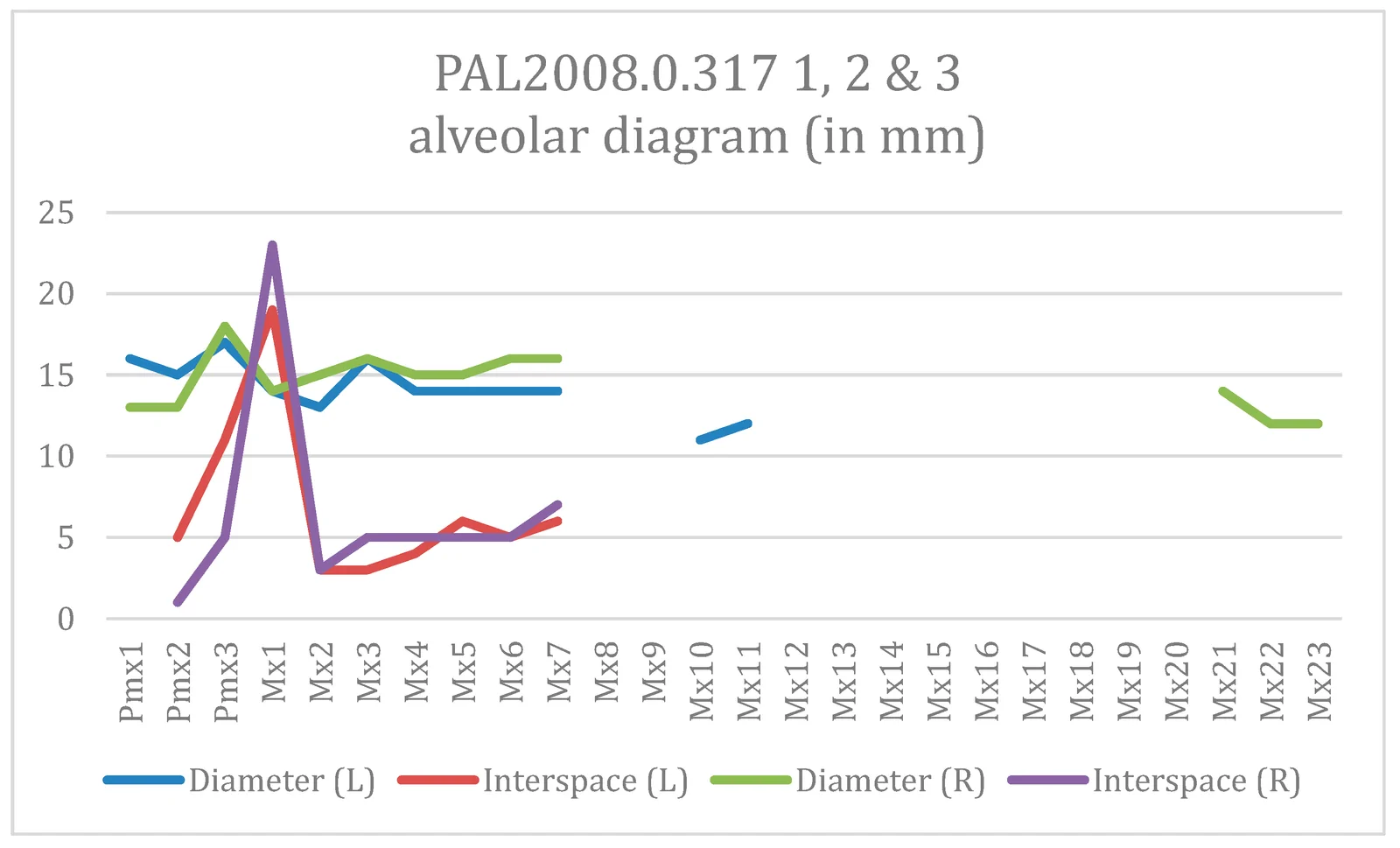
Alveolar diagram of *M. palpebrosus* PAL2008.0.317 1, 2 & 3 (Beauvoisine Museum, Rouen, France).

The alveolar diagram (Figure 5) shows the pseudo “heterodonty” of this specimen, with premaxillary teeth that are large compared to the maxillary teeth, which could be interpreted as giving the living animal a better grasp for catching prey. The premaxillary rows are asymmetrical, with the third left alveolus having a more anterior position, giving a diastema of 19 mm vs. 14 mm for the right row. The only teeth preserved are the “pathological” ones, and their aspect does not seem to differ from other Metriorhynchid teeth. All alveoli diameters are measured antero-posteriorly.

Ref. [16] cites a figure of 27 alveoli (3 pmx + 24 Mx) for the type specimen of *Metriorhynchus palpebrosus*.

### 2.4. Cranial Bones (Figure 1, Figure 2, Figure 3 and Figure 4)

#### 2.4.1. Dorsal View

Dorsally, the rostrum, at the level of the fused maxillaries, has a width of 6 cm, then 7 cm at the insertion of the nasals bone, increasing to 12.4 cm at the prefrontal insertion and reaching 17 cm (approx.) at their distal part. The bucco-dorsal height remains constant (5.2 cm) until mid-length of the nasal bone, where we observe an impressive elevation of the anteorbital area at the level of the 18th alveoli, giving the skull a “funnel” shape. This aspect is not biased by fossilization: the skull shows any distortion like all the skulls discovered in this area [7].

Premaxillaries: Both are preserved. Dorsally, they are classically metriorhynchid in form enclosing the external nare and posteriorly reaching the level of the fifth maxillary alveoli. They underline the nare, making the premaxillaries + the nare extend 5 mm beyond the lateral border defined by the dentaries. On the rostrum of *M. brevirostris* ([4] Figure 1), the premaxillary–maxillary complex has a tubular appearance, quite dissimilar to the spatulate form of our specimen. Moreover, while the authors acknowledge the historical restoration of the external nare, here it appears much more elongated. On the anterior border of the external nares, the premaxillary bones are not fused to each other. On the posterior border of each external nare, the base of a strong septum remains (9 mm width for each Pmx), suggesting a partial division of the external nare in the living animal.

In lateral view, the premaxillaries are clearly pending downwards compared to the maxillaries; this is quite different from the lateral view of the type species of *M. brevirostris* illustrated in Young et al.’s study [4] (Figure 2).

Ventrally, the premaxillaries (Figure 3B and Figure 4B) are asymmetrical, especially for the respective third alveolus. As already indicated, the incisive foramen is at the level of the second alveolus. Ventrally, the premaxillary–maxillary suture is located in the middle of the diastema and reaches the postero-medial edge of the third alveolus. It fuses with its equivalent along a sharp suture at the level of the incisive foramen. This seems to be identical to what has been observed in the holotype of *M. brevirostris,* but clearly the suture is more medial than that shown in Figure 8A of [4]: the suture does not extend as far as the alveolus, unlike in the illustration.

Length from antero-borders to incisive foramen: 3.2 cm;

Max width of the premaxillaries (at the level of the third alveolus): 62 cm;

Dorsal length: 17.1 cm;

Lateral maximal premaxillary convexity from the buccal plane: 0.5 cm.

Maxillaries: Both are preserved, but a striking feature is the marked dentition anomaly at the level of the 12th left maxilla. This alveolus is not orientated towards the buccal cavity but instead is oriented laterally. This is not a post-mortem feature: the alveolar border is clearly visible, and all posterior alveoli beyond this one are closed by bone, which is not the case with the right row. This is the first recorded case of this unusual pathology in Metriorhynchidae.

The right maxilla bears 24 alveoli. The cross-section of the maxillaries is elliptic, due to a slightly convex palatine face (H 5.2 cm × L 8.4 cm, at the level of the 14th right Mx).

The ornamentation, despite the age of the specimen, is not very pronounced, consisting of just slight longitudinal grooves.

Laterally, this bone is not scalloped by the edges of the alveoli: the lateral border appears relatively flat (max length laterally: 47.2 cm). Postero-laterally, this bone participates in the anterior border of the anteorbital fenestra, a classic feature among metriorhynchids [8].

Ventrally, the surface appears slightly convex. The tooth rows are not symmetrical, maybe due to the dentition anomaly of the 12th left. Moreover, the interalveolar space is not regular. The incisive foramen is located at the mid-level of the second Pmx alveolus. The insertion of the maxillary at this location is similar to the arrangement found in the Callovian species *Metriorhynchus brachyrhynchus* (Text-Figure 48 of [8]) and differs from *Metriorhynchus brevirostris*, where the insertion is at the level of the anterior part of the third alveolus as illustrated in [4] (Figure 1). Anteriorly, the premaxillary/maxillary diastema is well marked with the first two maxillary alveoli nearly coalescent.

No palatomaxillary grooves are visible, and the buccal face appears relatively flat (Figure 4B).

The insertion of the palatine bones is masked by oyster shell incrustations.

Nasals: These are difficult to observe due to the age of the specimen. Nevertheless, dorsally, they separate the maxillaries at the level of the eighth Mx alveolus. Their ornamentation is smooth and consists of longitudinal ridges.

Dorsally, the right nasal bone is fused to the prefrontal area 3.3 cm from the anterior part of the right prefrontal bone.

This area is highly variable intra- and interspecifically [20]. The anterior part of the frontal area seems to be inserted between the nasals at the level of insertion of the prefrontal bone. This feature is closely comparable to the callovian *Metriorhynchus leedsi* [8] (Text-Figure 73-A).

In the lateral view, the right side terminates at the level of the 23rd Mx alveolus, bordering the anteorbital fenestra dorsally. Laterally, and after the anteorbital fenestra, the prefrontal pillar elongates the nasal bone.

Prefrontal sections: Both are preserved but are deeply incrusted with serpulids and oysters. The dorsal ornamentation is not very pronounced except for the denticles of the external lateral ridges, overhanging the orbits. The relationship of the prefrontal to the frontal area can be also seen on the right side; an antero-posterior length of 10 cm and a maximum dorsal length of 5.5 cm can be estimated.

Laterally, the anteorbital pillar of the prefrontal area (Figure 3D and Figure 4D) is well separated from the visor-like dorsal edge of this bone (height 3.1 cm).

Jugal: This bone has been torn out on the right side; it was finger-like with a height of 1.2 cm. The left one is incrusted.

Lachrymal: The right one is well preserved and presents the notch of the posterior part of the anteorbital foramen. It forms the anteroventral border of the orbits.

Frontal: The most ornamented bone, which seems to be inserted between the nasal bones at the level of the prefrontal insertions.

Dorsally, from the anterior process part of the frontal bone between the nasal bones to the posterior suture with the parietal bone, a length of 17.2 cm with a width of 12 cm at the supra orbital notch (6 cm visible at left) could be estimated.

The antero-medial angle of the upper temporal fenestra is approximately 90°. The intertemporal ridge has a width of 1.9 cm and is fused to the parietal bone after 6.3 cm (total length of the intertemporal ridge: 14.3 cm).

In lateral view, the relationships to the parietal bone and laterosphenoid cannot be seen due to the closure of the sutures.

Ventrally, the base of the nasal sinuses can be seen up to the insertion of the laterosphenoid.

Parietal: This bone laterally separates the upper temporal fenestra and then divides to border each fenestra on the occipital view, giving the bone a Y-shape. The vertical bar of the “Y” is 1.9 cm long. The oblique branch is preserved over a length of 4.2 cm. The “Y” shape differs notably from the “T” shape of brevisrostrine forms like *M. hastifer* [20]. The lateral suture with the laterosphenoid cannot be seen due to oyster incrustations and the age of the specimen.

Squamosal: Dorsally, the insertion of the squamosal occurs 5 cm after the base of the oblique branch of the “Y” and prolongs it. Better preserved on the left side, it shows that the postero-lateral angle of the upper temporal fenestra has a strong notch, giving this fenestra a trapezoidal aspect. The width between the intertemporal pillar and the lateral border of the upper temporal fenestra is 10.5 cm.

Postorbital: This has virtually disappeared on both sides, with only a small part visible on the right side (Figure 4E).

#### 2.4.2. Palatine View

Palatine bones: As already mentioned, these are strongly incrusted with the shells of oysters. The only observation that can be made is that the total length is 6 cm at the infra-orbital region.

Pterygoids: Their posterior parts are preserved, but their relationships with the alisphenoid laterally and the vomer ventrally cannot be seen. The most striking feature is the V-shaped cross-section (preserved over a length of 10 cm for a proximal height of 5.7 cm), with an acute angle in the posterior view and not obtuse as in the Callovian *M. brachyrhynchus* ([8], Text-Figure 58).

Vomer: Only a sliver measuring 6 cm (L) × 7 cm (l) is preserved. It is visible from the distal part of the pterygoids to their distal end, at the basisphenoid (suture being invisible).

Basisphenoid: Again, due to the poor preservation and the age of the specimen, the sutures are difficult to discern. The ventral intertemporal bridge at this level is 2 cm in width.

Alisphenoid: The age of the specimen makes the relationships of these bones to others invisible.

#### 2.4.3. Occipital View (Figure 1E and Figure 2E)

Unfortunately, the state of preservation renders the auditory canal and all cranial foramina invisible.

The most striking feature of this occipital face is its height of nearly 14 cm from the cranial roof to the inner quadrate condyle.

The paroccipital bars and quadrates are well developed and overhang the occipital face.

Supraoccipitals: These bones do not participate in the foramen magnum, being excluded by the supraoccipital along 6 mm from the roof of the foramen. There are differences between this specimen and Andrew’s plate XIII of *M. “durobrivensis”*, which is considered to be *M. brachyrynchus* [20].

The first difference is that the supraoccipitals are not fused to each other along a ridge but on the reverse, along a small depression. The second is that the insertion on the exoccipitals is in a “V” shape with an acute angle rather than an obtuse angle.

Laterally, a small foramen can be seen along the insertion with the roof of the parietal.

Occipital height: 4.4 cm;

Occipital width: 1.8 cm.

Exoccipitals: There are no major differences from those already described [8]. Dorsally, they participate in the occipital condyle though a small lateral border. They are developed laterally, and the paraoccipital bars are prominent and terminate in a rough surface for muscular insertions, exceeding the occipital face by nearly 2.3 cm, the same level of the occipital condyle. Ventrally, after a notch, they fuse with the dorsal face of the quadrates as seen on the right side. As already mentioned, no nerve foramina can be seen. Their participation in the occipital condyle cannot be observed.

Maximum width from the roof of the foramen magnum to the lateral paraoccipital bars: 9.2 cm.

Basioccipital: This bone forms the occipital condyle. Again, the state of preservation does not allow us to see its relationship to the exoccipitals. It is well developed (l3.5 cm × h 2.8 cm). The internal carotid artery foramen is infilled by sediment. The basioccipital condyles are externally directed oblique ridges and are relatively well developed (L 3 cm × 18 cm).

Quadrate: Best preserved on the right side, this massive bone is strikingly identical to the description of *M. superciliosus* [8]. It ends with two convexities for the articulation with the mandibular condyle, the lateral convexity being more developed than the medial one. The preserved articular surface is 5.5 cm long.

Another striking feature is the size of this bone: the medial convexity overhangs the occipital plane by 5.6 cm, with the articular surface preserved over a length of 5.5 cm.

Width from foramen magnum to external condyle: 12.4 cm.

## 3. Discussion: Specific Attribution of This Specimen

### 3.1. The Genus Metriorhynchus

Recently, in 2021, the genus *Metriorhynchus* was reassessed [4] with a convincing demonstration on the validity *Metriorhynchus brevirostris*. This genus still has its holotype (MHNG V2232), a partial rostrum.

Based on this rostrum, a comparison is made with various historical *Metriorhynchus* rostra, in an attempt to demonstrate the variability of the maxillary–premaxillary suture among other *Metriorhynchus* taxa and some other metriorhynchid genera ([4], Figure 8). However, these comparisons are hampered by mistakes in identifying sutures, especially for the holotype (in [4], Figure 8A, the suture is clearly visible above the one underlined), or by errors in the view angle for the various taxa.

The phylogenetic analysis presented ([4], Figure 5) includes a cladogram that contains a number of problematic taxa. Furthermore, it compares the incomplete *M. brevirostris* rostrum with, among others, skulls sometimes involving entirely different regions of the skull.

The study by Young et al. advances their conclusion that the genus *Metriorhynchus* is polyphyletic and that the species currently included within this genus need to be redefined within a new genus, thus cutting the so-called “Gordian knot”.

Furthermore, closer examination of their cladogram reveals a number of strange taxa, including the following:

1. The “Chile Metriorhynchoid” and “Metriorhynchinae indet“ (Cuba): which species?
2. “Cretaceous rhacheosaurin” and “swiss rhacheosaurin”: comparison of a sub-family with species?
3. “cf. *Torvoneustes*”, “cf. *Cricosaurus*” and “*Torvoneustes* sp.”: if the genus is uncertain, why not use only the family, the higher taxon?
4. “Druegendorf Merge”: known as Geosaurini indet [21]; so only the family is defined and compared to a species?
5. “*Neptunidraco ammoniticus*”: the validity of this species is questionable (cf [11], its remains are too fragmentary (in a slice of stone)) as Geosaurinae and its stratigraphical level on a hard ground from this is dubious.
6. “Swiss snout”, “English snout”, “Mr Leeds Dakosaur” and “Mr Passmore specimen”: their “descriptions” only allow these specimens to be classified as indeterminate Metriorhynchidae (“E-Clade” near of Geosaurinae according to [21]).
  Despite [21] presenting a particular tooth morphology, this E-Clade has a specific rank given by the authors themselves. It shows a certain hesitation against mixing, especially against the tooth morphology plasticity among Metriorhynchids, as shown for our specimen.
7. “Vaches noires Dakosaur”: given that the Vache Noires levels are Callovo-Oxfordian, this Upper Jurassic–Cretaceous genus has never be identified there, OTU or not; it has no value.
8. “*Chouquet* cf. *hastifer*”: elsewhere in their study, this specimen is considered as *M. brevirostris*, while their cladogram seems to oppose this.
9. The specimen described here of *M. palpebrosus* is not present in their cladogram despite a discussion further in the same paper with its complete skull.

In short, out of the 48 taxa included in the cladogram, 15 cannot be used to compare a rostrum or other cranial part to a more complete skull at the species level.

Therefore, given that one-third of the specimens are irrelevant, can this cladogram be considered relevant?

Creating a new genus based on this analysis appears to be too hasty, so the Gordian knot remains despite the title of Young et al. ’s study.

The redefinition of the type species of *Metriorhynchus* as *M. brevirostris* respects the nomenclature code, but the redefinition of all other species based on this analysis will create a useless instability. As outlined in article 65.2 of the Zoological Code, such proposals must be motivated by the Zoological Commission to maintain stability as much as possible.

Cladistic analysis has to be considered as just one tool among others and not as the solution; it must be integrated with other tools like statistics, integrating the known variability.

For these reasons, we prefer to use the diagnosis of Vignaud [20], which takes into account the known variability within this genus, e.g., *M. superciliosus* (this paper and [22] in prep).

Concerning our specimen, three premaxillary teeth are present, the external nares are longitudinally orientated with a nasal septum, the prefrontal bones are well developed but not curved, the upper temporal fenestrae are sub-quadrangular, and the orbits are laterally oriented.

For all these reasons, this specimen is considered to be *Metriorhynchus Meyer* 1832 [15].

To try to solve the famous Gordian knot [4] of the genus *Metriorhynchus*, the solution lies in understanding the holotype itself in relation to its closest relatives from a taxonomical, stratigraphical (unfortunately at the at geological stage here) and geographical point of view.

### 3.2. Metriorhynchus brevirostris Versus European Kimmeridgian Species

Four *Metriorhynchus* species are present in the Upper Jurassic deposits of Europe ([4,20]):

*Metriorhynchus brevirostris* (Holl, 1829) [23].

*Metriorhynchus acutus* Lennier, 1887 [19].

*Metriorhynchus palpebrosus* (Philips, 1871) [16].

*Metriorhynchus hastifer* (Eudes-Deslongchamps, 1868) [7].

*Metriorhynchus brevirostris,* the holotype of the genus, is known only from a single rostrum and is well redescribed [4] but missing this essential characteristic underlined by [7] in 1867 and subsequently described in more detail in 1886 [19]. Nevertheless, the species remains valid and well defined.

*Metriorhynchus acutus* is more problematic. The type specimen is well illustrated and described but was destroyed in 1944 during the bombing associated with the “liberation of Le Havre”. Young et al. [4] consider it as another genus, *Gracilineustes*, but while agreeing with their arguments, with the absence of the skull, this conclusion is too speculative for the moment; the lack of bone ornamentation could be interpreted as the specimen being a juvenile of a bigger species.

*Metriorhynchus hastifer* is known from a specimen from the Lower Kimmeridgian of Cap de la Hève (Normandy, Northwest France) which was described successively by [7] and then [19] with nearly the same plates. It has been variously considered as a brevirostrine form [9]) and a longirostrine form ([10,20]). With an about 60% anteorbital region and with no contact between the premaxillary and frontal bones, it has to be considered as a mesorostrine form [18]. According to [7], this skull has 22 to 23 teeth.

Another specimen illustrated in [1], which comes from the Chouquet private collection, has been considered as *M.* cf. *hastifer*.

The last relatively well identified taxon is *Metriorhynchus palpebrosus* from the Kimmeridgian clay of Shotover Hill (England) (considered as Kimmeridgian [20] but may be Tithonian). This species is more longirostrine, with an elevated skull and sub-quadrangular upper temporal fenestrae; the elevation of the postorbital area is a striking feature among Metriorhynchids. With 66% of the anteorbital region, this skull clearly belongs to a longirostrine species. The number of upper teeth is about 23 or 24. Our specimen has an incomplete tooth formula, but the right row is similar.

The elevation of the skull that has led the specimen to be initially diagnosed as *M.* cf. *palpebrosus* ([1] and illustrated in [20], *Pers. Obs*. a cast is visible in the French national Museum of Paris).

The general description is similar and proportions to Philips’ description [16] and the [20] emended diagnosis; for this reason, this specimen is considered as *Metriorhynchus palpebrosus*. Nevertheless, the doubt on the exact stratigraphic position of our specimen in front of the age of the Shotover Hill deposits of the holotype has to be kept in mind.

Both specimens described in [1], *M.* cf. *hastifer* and *M. palpebrosus* skull were considered by Young et al. [4] as close to *M.* cf. *brevirostris* based on the shape of the tooth row, even though these authors did not observe the marked maxillary pathology of the Pouchet skull, which alters the left side but also impacts on the symmetry of the right row (aspect and alveolar interspace).

Regarding the skulls described herein, Young et al. [4] suggested that differences in the alveoli and tooth count could be due to individual, ontogenetic and/or sexual variation where dimorphism has never been formerly attested on metriorhynchids since [20].

These authors compare the incomplete rostrum of *M. brevirostris* to the complete skull of *M.* cf. *hastifer* (the Chouquet skull); they consider them to be taxonomically close without knowing if the postorbital area is elevated in “fumel” like *M. palpebrosus* or more flattened like *M. hastifer*.

For all these reasons, these determinations are not recognized.

At this stage, what is the exact status of these three Upper Jurassic species found in in France and England? And more especially, what is the status of the holotype of *Metriorhynchus brevirostris*, which consists of a single rostrum?

In fact, this question was already answered in the 19th century by authors who had the original material at hand. The answer is apparent in pl XXIV, Figure 4, in E. Eudes-Deslongchamps [7] and is re-illustrated by G. Lennier in pl XV, Figure 3 [19].

Both authors illustrated a cross-section of *M. hastifer* showing a curious feature, unique among *Metriorhynchus*; two longitudinal convex gutters on the buccal face of the rostrum behind the tooth row (Figure 4A). E. Eudes-Deslongchamps [7] described it briefly, but Lennier gave an extensive description, p. 74 [19], that could be translated as the following:

“The upper jaw clearly displays one of the most distinctive characteristics of *Metriorhynchus*: the median section situated between the two rows of alveoli—which is roughly flat or slightly convex in *Teleosaurus* = Metriorynchidae Sic)—features a broad, deep groove extending from one end to the other. Consequently, a cross-section of this area (Figure 3) reveals three longitudinal grooves: the first corresponding to the left row of alveoli, the second to the median groove, and the third to the right row of alveoli; two longitudinal ridges separate the broad median groove from the two alveolar grooves.”

Curiously, subsequent authors did not notice this characteristic because the feature was observed only on skulls that no longer exist.

Re-examining the Chouquet skull, *M.* cf. *hastifer*, it can be observed that this characteristic is clearly present. In contrast, concerning the same cross-section of de *M. palpebrosus*, that of our specimen at the same level is smooth like the buccal face of the *M. palpebrosus* holotype (or any other species of *Metriorynchus,* e.g., [8], or any Metryorhynchus species buccal face illustrated in [1]). This feature is present only in *M. hastifer*.

Based on this characteristic, *M. hastifer* cannot be assimilated to the same species as *M. palpebrosus* as proposed by [4].

Now, re-examining the photos of the holotype of *M. brevirostris*, it appears that we can see the same two longitudinal gutters as found in *M. hastifer* and described successively by Eudes-Deslongchamps and Lennier; this indicates that the rostrum of *M. brevirostris* could belong to the same species as *M. hastifer* and thus *brevirostris = hastifer*. The alveoli of *M. brevirostris* also appear to be more widely spaced than in the *M. hastifer* holotype but as shown by our specimen, the alveolar interspace could be variable due to ontogeny [20] or, like here, to pathology.

This is not a new idea. Ref. [19] (p. 71) already proposed that they were synonymous, but the idea was rejected due to the shape of the external nares [20].

This characteristic could serve as a “new” *Metriorhynchus* generic characteristic, but as underlined by [19], this is a specificity (in the literal sense, *cf* original comments above) of *M. brevirostris*: it has never been noticed in any another species of *Metriorhynchus,* from the Callovian to the Kimmeridgian (see buccal faces on [1,8,20]). It could be argued that this unique trait could be weak as the tooth morphology apparatus of the E-Clade [21] to define a taxon. But it has been noticed among nearly thousands of skulls, known only three times, and surely for the *Metriorhynchus* genus, even including before WWII destroyed beautiful specimens in Le Havre.

For these reasons, and moreover to avoid the risk of creating instability by defining a monospecific genus based only on this characteristic, it is considered as being of specific, as already outlined by [19], and not of generic value.

Due to junior synonyms, *M. brevirostris* is considered as the junior synonym of *M. hastifer*, a distinct species from *M. palpebrosus,* with these three longitudinal buccal ridges as a new specific characteristic.

The skull in the private collection of Mr. Chouquet may be the only known complete skull of *M. brevirostris* and may become an excellent paratype if it were located in a public collection and seriously studied. Having seen this specimen firsthand, the occipital face is crushed (see Figures 4 and 5 from [1]): a better preparation could validate (or not) our conclusions to see if this specimen belongs to the Metriorhynchus genus.

Coming back to our specimen, the “smooth” palatal face and the more detailed examination of the skull side by side with the holotype [1] have confirmed the initial diagnosis as *M. palpebrosus*.

To distinguish these species, and given that the genus *Metriorhynchus* is no longer monospecific, the following emended diagnosis is proposed, based on the combined studies of [4,10,20].

### 3.3. European Metriorhynchus Species Taxonomy

Order: Mesoeucrocodylia Whestone and Whybrow, 1983 [12];

Sub-order: Thalattosuchia Fraas, 1901 [13];

Family: Metriorhynchidae Fitzinger, 1843 [14];

Genus: *Metriorhynchus* von Meyer, 1832 [15];

Type species: *Metriorhynchus brevirostris* (Holl, 1829) [23].

Taking into account that *Metriorhynchus brevirostris* is no longer known only from an isolated rostrum and that the Metriorhynchus genus is not monospecific, the following diagnosis, combining various studies [4,17,20], is proposed:

Emended diagnosis of the genus:

Premaxillaries are fused in a posterodorsal process that terminates at the level of the M3 alveoli; the three premaxillary alveoli successively increase in size, with constriction at the premaxilla–maxilla contact; having at least 13 maxillary alveoli anterior to the palatine anterior processes; nasal bone anterior processes terminate level with the M8 alveoli; lacks a fully ossified internarial bar (thus an undivided external naris); the anterior margin of the narial fossa is posterior to the P1 alveoli; external nare is directed upward. Prefrontal areas are well developed but not curved downward. Postorbital bar is perpendicular to the medial plane of the skull. Supratemporal fenestrae are sub-quadrangular. On the occipital face, the carotidian formina is open and not protected. Choanae are always separated by a medial septum. Pterygoidal fossa is closed posteriorly.

The teeth have less pronounced, slightly compressed carinae, depending on the position on the tooth row and the age of the specimen.

Concerning *M. brevirostris*, the diagnosis used by [4] is emended by including the diagnosis of *M. hastifer*, a junior synonym, and the additional buccal characteristics [20] (the two parallel maxillary gutters).

#### 3.3.1. *Metriorhynchus brevirostris* (Holl, 1829) [23]

1820: Upper jaw of the fossil crocodile from Le Havre in the Museum of the Academy of Geneva—De la Beche, lithograph [24].

1824: Head with a shorter snout [partim]—Cuvier, pp. 152–153, pl. 10, Figures 5–7 [25].

1829: *Steneosaurus brevirostris* sp. nov.—Holl, p. 88 [23].

1831: *Gavialis Jurinii* sp. nov.—Gray, p. 57 [sic] [partim] [26].

1832: *Metriorhynchus geoffroyii* gen. & sp. nov.—von Meyer, p. 106 [partim] [15].

1836: Anterior extremity of the upper jaw of Steneosaurus—Buckland, p. 36, pl. 25, Figure 3 [27].

1837: *Metriorhynchus Geoffroyii* (von Meyer)—Bronn, p. 520, pl. 26, Figures 7b,d and 8a,b [sic] [partim] [28].

1845: *Steneosaurus rostro-minor* [sic] Geoffroy Saint-Hilaire—Pictet, p. 46, pl. 1, Figure 2 [partim] [29].

1853: *Steneosaurus rostro-minor* [sic] Geoffroy Saint-Hilaire—Pictet, p. 492–493, pl. 25, Figure 9 [partim] [30].

1868: *Teleosaurus hastifer* [sic]—Eudes-Deslongchamps, p. 112–118 [7].

1869: *Teleosaurus hastifer* Eudes-Deslongchamps [sic]—E. Eudes-Deslongchamps, 124–221 [7].

1867–1869: *Metriorhynchus hastifer* (Eudes-Deslongchamps) [sic]—E. Eudes-Deslongchamps, p. 343, pl. 23 Figure 2, pl. 24, Figures 1–5 [7].

1886: *Metriorhynchus hastifer* (Eudes-Deslongchamps) [sic]—Lennier, p. 71–80, pl. 15, Figures 1–4, pl. 16, Figures 1 and 2 [19].

1900: *Metriorhynchus hastifer* (Eudes-Deslongchamps) [sic]—Sauvage, p. 498–499 [31].

1936: *Metriorhynchus hastifer* (Eudes-Deslongchamps) [sic]—Kuhn, p. 66–67 [32].

1939: *Metriorhynchus hastifer* (Eudes-Deslongchamps) [sic]—Bigot, p. 26 [33].

1973: *Metriorhynchus geoffroyi* (von Meyer) [sic]—Steel., p. 45, Figure 18 (10) [34].

1973: *Metriorhynchus hastifer* (Eudes-Deslongchamps) [sic]—Steel, p. 46 Figure 19 [34].

1982: *Metriorhynchus hastifer* (Eudes-Deslongchamps) [Sic]—Buffetaut, P. 26 [10].

1987: *Metriorhynchus geoffroyi* (von Meyer) [sic]—Adams-Tresman, p. 193. [35].

1995: *Metriorhynchus geoffroyi* (von Meyer)—Vignaud, p. 218–219 [20].

1995: *Metriorhynchus geoffroyi* (von Meyer) [Sic]—Vignaud, p. 225–226 [20].

2008: *Metriorhynchus* cf. *hastifer* (Eudes-Deslongchamps) [Sic]—Lepage et al., p. 50–56 [1].

2021: *Metriorhynchus brevirostris* (von Meyer) [sic]-[4].

Holotype: MHNG V-2232, partial cranial rostrum.

Casts of holotype: MGCL 9868, PIMUZ A/III 82 and OUMNH unnumbered.

Type locality and horizon: Le Havre, Department of Seine-Maritime, Upper Normandy, France.

Kimmeridgian, Upper Jurassic.

Emended diagnosis:

Premaxillary posterodorsal processes terminate at the level of the M3 alveoli; maxillary alveoli M1–M13 are irregularly shaped ovals, with the post-M3 alveoli becoming more circular in shape; nasal bone anterior processes terminate level with the M8 alveoli; the anterior margin of the narial fossa is posterior to the P1 alveoli, and the posterior margin terminates slightly posterior to the premaxillary tooth row. The snout is stubby with elongated nasal bones; the prefrontal bones are slightly developed, quadrangular; the frontal bone is gracile. Tooth alveoli (22–23 upper row) have a regular diameter and are close to each other. 

Autapomorphic feature: n the palatal view, the buccal area is showing two convex gutters (one parallel to each tooth row).

#### 3.3.2. *Metriorhynchus palpebrosus* (Philips, 1871) [16]

1859: Tête presqu’entière d’une sorte de crocodile fossile—Pouchet, p. 44 [2].

1871: *Steneosaurus palpebrosus* nov. sp.—Philips, p. 380–384, Figures 182–187 [16].

1890: *Metriorhynchus palpebrosum* (Philips)—Woodward & Sherbon, p. 250 [36].

1936: *Metriorhynchus palpebrosus* (Philips)—Kuhn, p. 65 [32].

1973: *Metriorhynchus palpebrosus* (Philips)—Steel, p.48 [34].

1982: *Metriorhynchus palpebrosus* (Philips)—Buffetaut, p. 26 [10].

1995: *Metriorhynchus palpebrosus* (Philips)—Vignaud, p. 226–227 [20].

2008: *Metriorhynchus* cf. *palpebrosus* (Philips)—Lepage et al., p. 136–139 [1].

2021: *Metriorhynchus* cf *brevirostris* (von Meyer) [sic]—Young et al., 2021, p. 546 [4].

Holotype: skull + half mandible, OUM J 2,9823.

Type locality: Shotover Hill (Oxfordshire, UK), Cap de la Hève (Normandy, France), Kimmeridgian.

Emended diagnosis (a combination of [20] and the present study):

The fronto-parietal bar is well marked. Anteorbital fenestra is well developed. Prefrontal bone is well developed. Occipital reliefs are pronounced with exoccipitals overhanging the level of the occipital condyle.

Autapomorphic characters: Tooth alveoli are well developed, rounded and well separated (23–24/17–19). The snout represents 65 to 70% of anteorbital area, The skull is massive with a postorbital area that is blunt and elevated, in “fumel”, in frontal view. The snout has an elliptical cross-section with a smooth buccal face. Sub-quadrangular supratemporal fenestra.

## 4. Conclusions

This skull is the first *Metriorhynchus palpebrosus* described in France, showing a unique pathology never seen before among metriorhynchids. This species has been poorly described before, and this study provides a better interpretation of this species.

This study helps in the definition of the genus *Metriorynchus* based on the rediscovery of a forgotten characteristic, the “double buccal gutters”, relative to the morphology of the buccal area, which was used by earlier authors but has been overlooked since 1886.

The very important snout of *Metriorhynchus brevirostris* described by Young et al. [4], the basis of the *Metriorhynchus* genus and then the Metriorhynchidae family, was identified as an isolated species due to its partial state. It was improbable that this species was not described before due to the number of all other skulls found from even the Callovian to the Kimmeridgian from Europe ([1,4,7,8,20] among other studies of European collections from firsthand studies). This character finally allows us to find a good candidate to associate more complete specimens, identified previously as *M. hastifer*, to the type species *M. brevirostris*.

It also helps to put *M. hastifer* in synonymy with *M. brevirostris*. As usual, the discovery of new material provides better understanding of taxonomical problematics like the “Gordian knot” of *Metriorhynchus*.

The skull and skeleton in the private collection of Mr. Chouquet, the only complete *M. brevirostris* skull known, could make an excellent paratype if they are one day housed in a public collection.

A direct consequence of a better definition of the type species of *Metriorhynchus* is that it leads to a better definition of the coexisting species like *M. palpebrosus*.

## Data Availability

The original contributions presented in this study are included in the article. Further inquiries can be directed to the corresponding author.
